# Cubic and quadratic distortion products in vibrations of the mouse cochlear apex

**DOI:** 10.1121/10.0015244

**Published:** 2022-11-21

**Authors:** James B. Dewey

**Affiliations:** Caruso Department of Otolaryngology—Head & Neck Surgery, University of Southern California, Los Angeles, California 90033, USA jamesdew@usc.edu

## Abstract

When the ear is stimulated by two tones presented at frequencies *f*_1_ and *f*_2_, nonlinearity in the cochlea's vibratory response leads to the generation of distortion products (DPs), with the cubic 2*f*_1_–*f*_2_ DP commonly viewed as the most prominent. While the quadratic *f*_2_–*f*_1_ DP is also evident in numerous physiological and perceptual studies, its presence in the cochlea's mechanical response has been less well documented. Here, examination of vibratory DPs within the mouse cochlea confirmed that *f*_2_–*f*_1_ was a significant and sometimes dominant component, whether DPs were measured near their generation site, or after having propagated from more basal locations.

## 1. Introduction

**Fig. 1.**
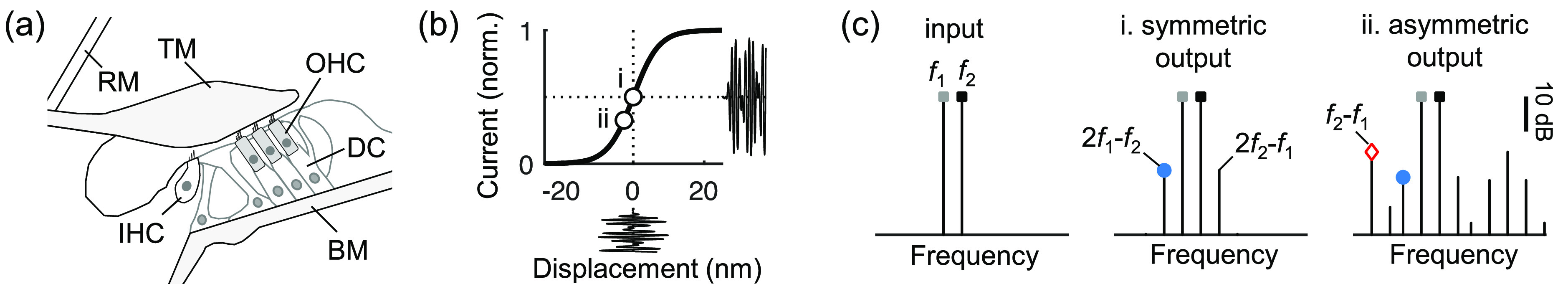
Dependence of even- and odd-order DPs on OHC nonlinearity. (a) Schematic cross section of the organ of Corti (DC, Deiters' cell; IHC, inner hair cell; RM, Reissner's membrane). (b) First-order Boltzmann function used to approximate the nonlinear relationship between OHC stereociliary bundle displacement and transduction current. For a given displacement *x*, the output current *I* is given by $I\left(x\right)=\frac{1}{\left.1+e^{a_{1}\left(x_{1}-x\right)}\right.},$ where *a*_1_ determines the slope (here, *a*_1_ = 0.28 nm^−1^) and *x*_1_ sets the OP. Waveforms for a two-tone input and the output when the OP is at the center of the function (*x*_1_ = 0) are shown. (c) Spectra of the function's two-tone input and its output when the OP is centered (i), resulting in a symmetric output and only odd-order DPs (e.g., 2*f*_1_–*f*_2_ and 2*f*_2_–*f*_1_), or uncentered (ii; *x*_1_ = 2.6 nm), resulting in an asymmetric output and additional even-order DPs (e.g., *f*_2_–*f*_1_).

In the mammalian cochlea, sound-evoked waves traveling along the basilar membrane (BM) are actively amplified by the outer hair cells (OHCs) within the organ of Corti [Fig. 1(a)]. The amplification process is highly nonlinear, resulting in phenomena like compression, suppression, and distortion in the cochlea's mechanics (Robles and Ruggero, 2001). For instance, in response to two stimulus tones at frequencies *f*_1_ and *f*_2_ (*f*_2_ > *f*_1_), the cochlea generates significant intermodulation distortion products (DPs) at frequencies such as the “cubic” difference tone 2*f*_1_–*f*_2_ and the “quadratic” difference tone *f*_2_–*f*_1_. These distortions shape the input to the inner hair cells, and thus, the responses of the afferent auditory nerve, and are ultimately perceived (Goldstein, 1967; Humes, 1980; Plomp, 1965). However, the precise nature of the underlying nonlinearity and how it influences the cochlea's output remain incompletely understood.

Nonlinearity in cochlear mechanics is largely thought to be due to the saturating, sigmoidal relationship between deflection of the OHC's stereociliary bundle and the currents that flow through mechanically gated channels located near the stereocilia's tips (Avan *et al.*, 2013) [Fig. 1(b)]. Transduction currents produce the variations in membrane potential (i.e., the receptor potential) that drive electromotile force generation by the OHCs (Brownell *et al.*, 1985; Santos-Sacchi and Dilger, 1988). The resting position of the bundle, or its operating point (OP), is of primary interest as it determines the amplificatory gain provided by the OHC, as well as the relative magnitudes of any even- and odd-order DPs (e.g., *f*_2_–*f*_1_ and 2*f*_1_–*f*_2_, respectively) in its motile response. If the OP is near the center of the function, where the gain is highest, two-tone stimulation elicits symmetric currents that primarily contain odd-order DPs [Fig. 1(c)]. Any bias away from the center results in asymmetric currents and the presence of even-order components.

Though 2*f*_1_–*f*_2_ is the most readily perceived DP (Goldstein, 1967) and is generally the largest DP emitted to the ear canal, sizable responses at *f*_2_–*f*_1_ have been observed in auditory nerve fiber recordings (Kim *et al.*, 1980) and intracochlear or intracellular potentials (Cheatham and Dallos, 1997; Gibian and Kim, 1982; Nuttall and Dolan, 1993). However, the presence and relative magnitude of *f*_2_–*f*_1_ in the cochlea's mechanics have been less characterized. While *f*_2_–*f*_1_ is small or absent in BM vibrations measured from the cochlear base in guinea pig and chinchilla (Nuttall and Dolan, 1993; Rhode, 2007; Robles *et al.*, 1997), it has been observed in vibrations of the tectorial membrane (TM) in apical, low-frequency regions (Cooper and Rhode, 1997). Recent measurements from the gerbil base using low-coherence heterodyne interferometry (Ren and He, 2020) and optical coherence tomography (OCT; Vavakou *et al.*, 2019) have also found *f*_2_–*f*_1_ in vibrations of the OHC region—the presumed source of the DPs—although they appear to be smaller than 2*f*_1_–*f*_2_ (Burwood *et al.*, 2022).

Here, OCT was used to compare *f*_2_–*f*_1_ and 2*f*_1_–*f*_2_ DPs in vibrations from the 9 kHz location in the mouse cochlear apex. DPs were characterized both locally near where they are generated, including within the OHC region, and after having propagated from more basal generation sites.

## 2. Methods

Measurements were obtained from 11 adult (4–7 week-old) CBA/CaJ mice (five female) using a custom-built, swept-source OCT system and methods largely described in Dewey *et al.* (2021). All procedures were approved by the University of Southern California's Institutional Animal Care and Use Committee.

Mice were anesthetized (80–100 mg/kg ketamine; 5–10 mg/kg xylazine), placed on a heating pad (38 °C), and fixed to a head-holder. An otoacoustic emission probe (ER-10X; Etymotic Research, Elk Grove, IL) was sealed over the resected ear canal to present acoustic stimuli. Stimulus levels were calibrated using the pressure measured by the probe, which was corrected for the probe's frequency-dependent sensitivity.

After surgically accessing the left middle ear space, the OCT light source was scanned across the cochlea to obtain two-dimensional cross-sectional images of the apical turn. Vibratory responses to single- and two-tone stimuli were then obtained from the OHC region (close to the DCs), BM, and/or TM, with responses sampled at 100 kHz. Stimuli were 102 ms tones (with 1 ms ramps) presented 8–32 times with a ∼7 ms interstimulus interval. Single-tone responses were used to determine the measurement site's characteristic frequency (CF), which was defined as the frequency eliciting the largest BM displacement for tones presented at 30 dB sound pressure level (SPL). Measurements were only obtained from locations with a CF of 9 kHz. Various two-tone paradigms for measuring DPs are described in Sec. 3.

After the desired measurements were performed, mice were euthanized by anesthetic overdose. Certain measurements were repeated postmortem to verify the physiological origin of the DPs, which were greatly reduced or absent after death. Acoustic distortion was sometimes still detected in the ear canal at high stimulus levels, though it was at least 60–70 dB lower than the stimulus levels. Any acoustic distortion capable of eliciting a displacement as large as that observed in the *in vivo* measurements was considered problematic, and data collected using such stimulus conditions were not included in any analyses or plots.

Magnitudes and phases of responses at *f*_1_, *f*_2_, *f*_2_–*f*_1_, and 2*f*_1_–*f*_2_ were obtained by applying a fast Fourier transform to the steady-state portion of the average displacement waveform. Reported displacement magnitudes are root mean square values and phases of the acoustic stimuli have been subtracted from the displacement phases. For *f*_2_–*f*_1_ and 2*f*_1_–*f*_2_ DPs, this involved subtracting *φ*_2ec_–*φ*_1ec_ and 2*φ*_1ec_–*φ*_2ec,_ respectively, where *φ*_1ec_ and *φ*_2ec_ were the phases at *f*_1_ and *f*_2_ in the ear canal. Noise floors for each response component were calculated as the mean + 3 standard deviations of the displacement magnitudes within 220–320 Hz (for *f*_1_ and *f*_2_) or 20–120 Hz (for the DPs) of the response frequency. Unless noted otherwise, only data with magnitudes exceeding the noise floor are plotted, and averages are only shown when such data were available from at least three mice. All individual data are accessible in an online repository.^1^

## 3. Results

**Fig. 2.**
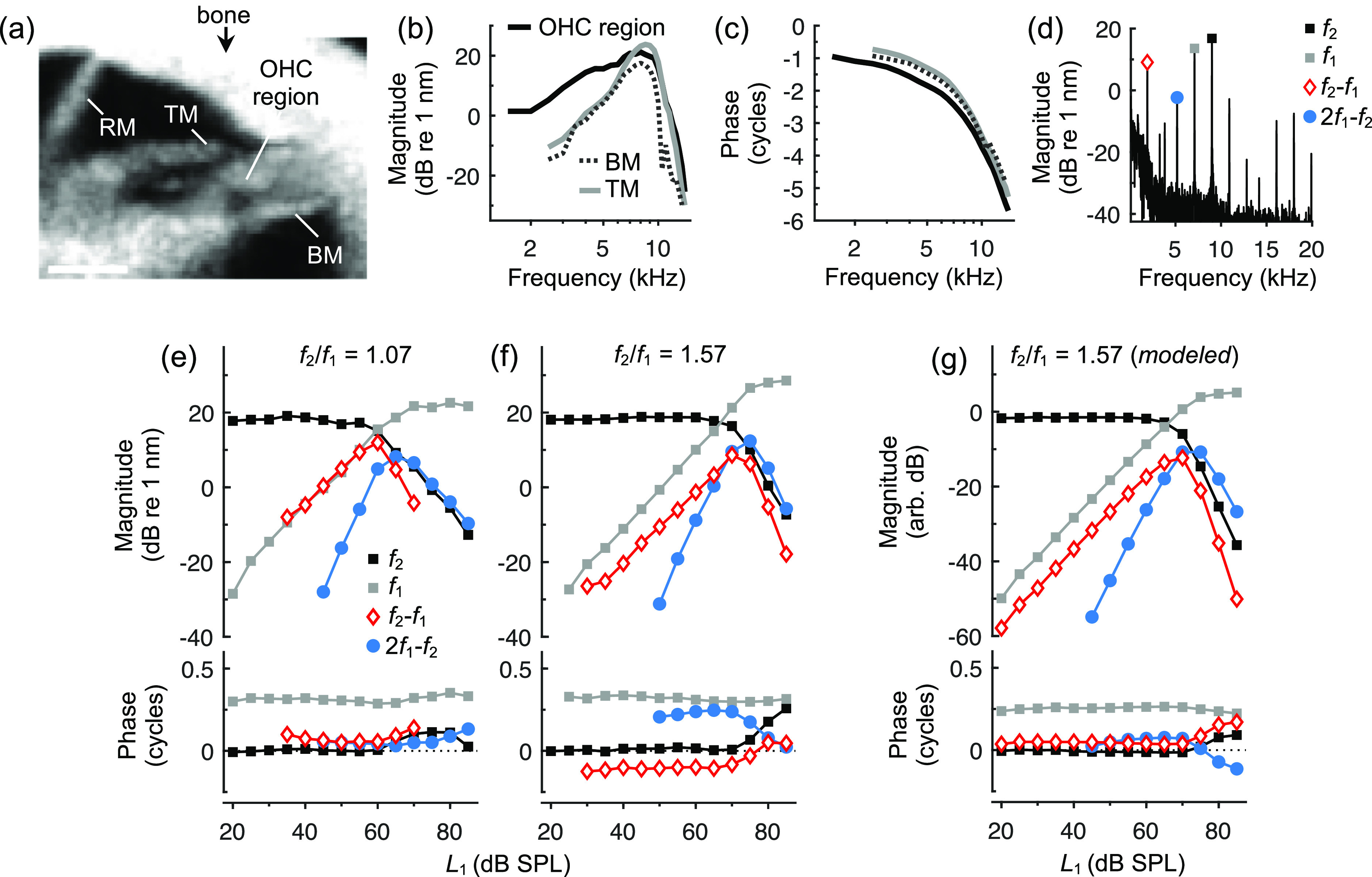
Single- and two-tone vibratory responses from the mouse cochlear apex. (a) OCT image of the apical turn with relevant anatomy indicated. Scale bar = 100 um. Magnitudes (b) and phases (c) of OHC region, BM, and TM displacement responses to 60 dB SPL tones varied from 1–15 kHz in an individual mouse. (d) Representative spectrum of the OHC region response to two 60 dB SPL tones (*f*_2_ = 9 kHz, *f*_1_ = 7.09 kHz), with responses at *f*_1_, *f*_2_, *f*_2_–*f*_1_, and 2*f*_1–_*f*_2_ indicated. (e) and (f) Magnitudes and phases of representative OHC region displacements as a function of *L*_1_ (with *L_2_* = 60 dB SPL) for two *f*_2_/*f*_1_ ratios and *f*_2_ = 9 kHz. Phases were referenced to the median phase of the *f*_2_ response for *L*_1_ < 40 dB SPL. Due to the higher measurement noise at low frequencies, lower-frequency DPs only became detectable when they were large (e.g., for *f*_2_/*f*_1_ = 1.07, *f*_2_–*f*_1_= 0.59 kHz while 2*f*_1_–*f*_2_ = 7.82 kHz; in contrast, for *f*_2_/*f*_1_ = 1.57, *f*_2_–*f*_1_ = 3.27 kHz and 2*f*_1_–*f*_2_ = 2.46 kHz). (g) Modeled responses for *f*_2_/*f*_1_ = 1.57 using the Boltzmann function shown in Fig. 1(b) with uncentered OP (see main text).

OCT was used to image the mouse cochlear apex [Fig. 2(a)] and measure vibratory responses to single- and two-tone stimuli from the OHC region, BM, and TM. Responses to single tones were tuned to a CF of 9 kHz and exhibited increasing phase lags with frequency that are indicative of traveling wave propagation [Fig. 2(b) and 2(c)]. As shown previously (Dewey *et al.*, 2021), the OHC region was more responsive to low frequencies compared to the BM and TM, which were more sharply tuned. The low-pass nature of the OHC region motion likely reflects the more direct influence of electromotility, which is thought to inherit a low-pass characteristic due to filtering of the receptor potential by the OHC's electrical properties (Santos-Sacchi, 1989; Vavakou *et al.*, 2019).

After determining the site's CF, OHC region responses to two-tone stimuli were obtained with *f*_2_ fixed at the CF and *f*_1_ varied to achieve *f*_2_/*f*_1_ ratios of ∼1.07–1.67 in 0.1 steps. As shown in Fig. 2(d), OHC region displacement spectra revealed numerous DPs, most prominent typically being *f*_2_–*f*_1_, followed by 2*f*_1_–*f*_2_. The presence and relative magnitude of *f*_2_–*f*_1_ were therefore consistent with the output of a Boltzmann function with an OP positioned away from the function's center [e.g., Fig. 1(c)].

To better characterize the underlying nonlinearity, measurements were made with *L*_2_ fixed at 60 dB SPL and *L*_1_ varied from 20 to 85 dB SPL [Fig. 2(e) and 2(f)]. For both small and large *f*_2_/*f*_1_ ratios [Figs. 2(e) and 2(f), respectively], OHC region displacements at *f*_2_–*f*_1_ and 2*f*_1_–*f*_2_ exhibited nonmonotonic growth patterns that were tied to the magnitudes of the responses at *f*_1_ and *f*_2_. For *L*_1_ values where the response at *f*_1_ remained smaller than the response to *f*_2_, the *f*_2_–*f*_1_ DP was larger than 2*f*_1_–*f*_2_ but grew less steeply with *L*_1_ (at a rate of ∼1 dB/dB, compared to ∼2 dB/dB for 2*f*_1_–*f*_2_). The different growth rates were consistent with the output of a power-law nonlinearity (e.g., Humes, 1980). For small ratios, the *f*_2_–*f*_1_ DP could even be as large as the *f*_1_ response, despite *f*_2_–*f*_1_ being far below the CF (e.g., *f*_2_–*f*_1_ = 0.59 kHz when *f*_2_/*f*_1_ = 1.07). As *L*_1_ was increased so that the response at *f*_1_ approached and then exceeded that at *f*_2_ (which became suppressed by the *f*_1_ response), both *f*_2_–*f*_1_ and 2*f*_1_–*f*_2_ DPs peaked and then rapidly declined. Because the 2*f*_1_–*f*_2_ DP started to decline at slightly higher *L*_1_ values, it typically became larger than the response at *f*_2_–*f*_1_ as *L*_1_ was increased further. DP phases were relatively constant for *L*_1_ < 60 dB SPL but could shift by up to 0.25 cycles at higher levels. The magnitude and direction of these shifts were predictable from changes in the phases of the *f*_1_ and *f*_2_ responses. Specifically, they were consistent with the phases of *f*_2_–*f*_1_ and 2*f*_1_–*f*_2_ being *φ*_2_-*φ*_1_ and 2*φ*_1_–*φ*_2_ (plus some constant), where *φ*_1_ and *φ*_2_ are the *f*_1_ and *f*_2_ response phases.

While these magnitude and phase patterns may appear complex, they were replicated by the output of the Boltzmann function shown in Fig. 1(b) when the OP was uncentered (*x*_1_ = 2.6 nm). Figure 2(g) shows the Boltzmann's output for *f*_2_/*f*_1_ = 1.57 when using BM displacements at *f*_1_ and *f*_2_ as the function's inputs (measured using the same stimulus paradigm and averaged from five mice). The Boltzmann's output was low-pass filtered (first-order, corner frequency = 1.75 kHz) in order to approximate filtering of the OHC receptor potential. Such filtering was previously found necessary to account for the relative magnitudes of harmonic and tonic distortions in single-tone responses (Dewey *et al.*, 2021), and can explain the large *f*_2_–*f*_1_ DP magnitude at small *f*_2_/*f*_1_ ratios, where *f*_2_–*f*_1_ falls below the corner frequency. While DPs in the Boltzmann's output also exhibited level-dependent phase shifts, the absolute phases of the modeled and measured DPs differed somewhat. This is not surprising, as vibratory phases change rapidly within the OHC region (Dewey *et al.*, 2021) and are undoubtedly influenced by mechanical properties not included in the Boltzmann model.

**Fig. 3.**
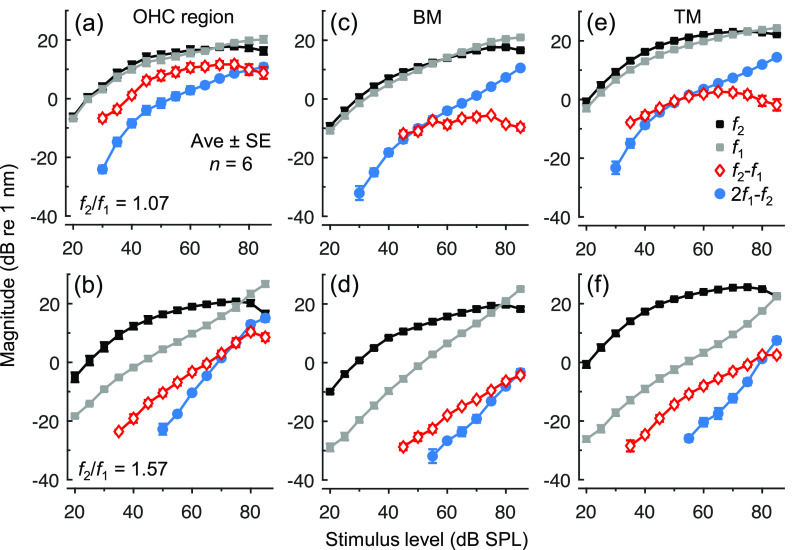
Comparison of DPs in OHC region, BM, and TM vibrations for equal-level stimuli. (a–f) Average (*n *=* *6) magnitudes of OHC region (a, b), BM (c, d), and TM (e, f) displacements at *f*_1_, *f*_2_, *f*_2_*–f*_1_, and 2*f*_1_–*f*_2_ as a function of stimulus level, with *f*_2_ = 9 kHz and *f*_2_/*f*_1_ = 1.07 (a, c, e) or 1.57 (b, d, f). Error bars indicate 1 SE and are often smaller than the symbols.

The level-dependent growth of OHC region DPs was further explored using equal-level stimuli, as shown for small and large *f*_2_/*f*_1_ ratios in Fig. 3(a) and 3(b). With *L*_1_ = *L*_2_, DPs generally grew less steeply with increasing level compared to when *L*_2_ was fixed and *L*_1_ was varied. When averaged across all *f*_2_/*f*_1_ ratios and mice, and evaluated for *L*_1_ = 40–55 dB SPL, growth rates for *f*_2_–*f*_1_ were an average (± standard error, SE) of 0.76 ± 0.06 (*n *=* *6) and 0.92 ± 0.02 (*n *=* *7) dB/dB for equal-level and fixed-*L*_2_ paradigms, respectively. For 2*f*_1_–*f*_2_, these rates were 0.96 ± 0.05 and 1.89 ± 0.05 dB/dB. The lower growth rates for the equal-level paradigm can be attributed to the compressive growth of responses at both *f*_1_ and *f*_2_ when *L*_1_ and *L*_2_ are covaried. The behavior of the DPs was otherwise similar between paradigms, with the 2*f*_1_–*f*_2_ DP growing more steeply than *f*_2_–*f*_1_ and becoming larger only when the *f*_1_ response exceeded the *f*_2_ response.

Equal-level stimuli yielded measurable DPs over a wide range of stimulus levels and were therefore also used to examine DPs in vibrations of the BM and TM [Figs. 3(c)–3(f)]. DPs were measurable from both structures though were typically much smaller than the OHC region DPs (by ∼10–20 dB and ∼5–10 dB for the BM and TM, respectively). The relative magnitudes of *f*_2_–*f*_1_ and 2*f*_1_–*f*_2_ DPs in BM and TM vibrations also depended strongly on the *f*_2_/*f*_1_ ratio, with *f*_2_–*f*_1_ being particularly reduced at small ratios [Figs. 3(c)–3(e)]. At these ratios, *f*_2_–*f*_1_ becomes very low in frequency while 2*f*_1_–*f*_2_ approaches the CF. The relative DP magnitudes therefore appear to be shaped by the frequency responses of the BM and TM, which are both sharply tuned to the CF. For *f*_2_/*f*_1_ ratios > 1.5 [e.g., Figs. 3(d) and 3(f)], *f*_2_–*f*_1_ is higher in frequency than 2*f*_1_–*f*_2_ and therefore does not suffer from this relative attenuation, explaining why it remained the dominant DP on the BM and TM.

**Fig. 4.**
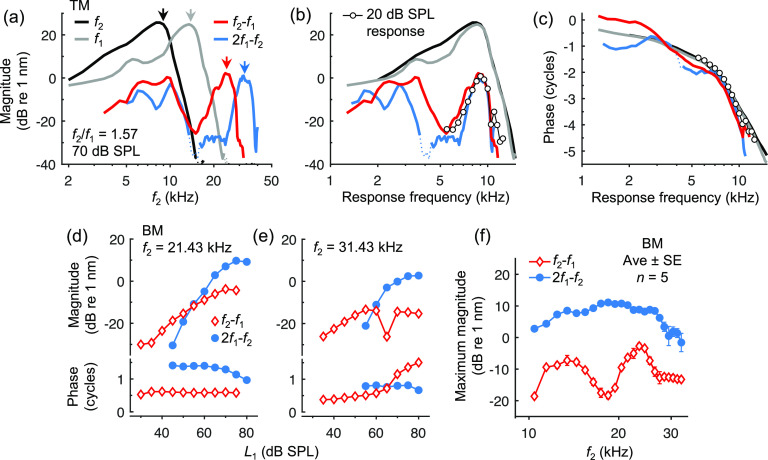
Propagation of DPs to the 9 kHz location. (a) Representative TM displacements vs *f*_2_ with *f*_2_/*f*_1_ = 1.57 and *L*_1_ = *L*_2_ = 70 dB SPL. Arrows indicate when each component frequency was equal to 9 kHz. The dotted portion of the curve for 2*f*_1_–*f*_2_ indicates where data fell below the noise floor. (b) Same as in (a) but with components plotted vs their own frequency. TM responses to individual 20 dB SPL tones are shown for comparison. (c) Phases of the responses in (b). (d, e) BM response magnitudes and phases at *f*_2_*–f*_1_ and 2*f*_1_–*f*_2_ with *f*_2_ and *f*_1_ set so that the DP frequencies were equal to 9 kHz, with *L*_2_ = 60 dB SPL and *L*_1_ varied. Data from one mouse are shown for two *f*_2_ frequencies. (f) Average (*n *=* *5) maximum DP magnitudes obtained with the paradigm used in (d, e) as a function of *f*_2_.

Both DPs were also measurable at the 9 kHz location after having been generated at more basal sites and then propagated apically. Figure 4(a) shows TM responses obtained with *f*_2_ varied from ∼2–40 kHz, *f*_2_/*f*_1_ = 1.57, and *L*_1_ = *L*_2_ = 70 dB SPL, plotted vs the *f*_2_ frequency. DP magnitudes peaked when *f*_2_ was near the CF, where there was maximal interaction between the responses at *f*_1_ and *f*_2_, as well as when the DP frequency fell near the CF (see arrows). This occurred when there was little local interaction between the responses at *f*_1_ and *f*_2_, which peaked at more basal sites. The measured DPs therefore presumably originated at these sites and propagated to the 9 kHz location. When plotted vs their own frequency, DP magnitudes and phases for frequencies > 6 kHz resembled those of responses to single tones presented at 20 dB SPL [Figs. 4(b) and 4(c)]. Phases of lower-frequency DPs were more complex, possibly indicating the presence of both locally generated and apical- or basal-propagating components (Dong and Olson, 2008).

For large *f*_2_/*f*_1_ ratios, propagated *f*_2_*–f*_1_ DPs were often greater in magnitude than 2*f*_1_*–f*_2_ DPs, particularly for lower-level stimuli. With *f*_2_/*f*_1_ = 1.57 and stimuli presented at 60 dB SPL, propagated *f*_2_*–f*_1_ and 2*f*_1_*–f*_2_ DP magnitudes on the TM were on average (± SE) 0.36 ± 0.06 nm and 0.18 ± 0.05 nm, respectively (*n *=* *5), equivalent to displacements elicited by a 9 kHz tone at ∼11 and 6 dB SPL. However, these comparisons are complicated by the fact that higher *f*_2_ frequencies were required to generate 2*f*_1_*–f*_2_ at 9 kHz. Cochlear sensitivity, nonlinearity, and responsiveness to force generation at 9 kHz may all vary with location, potentially contributing to the different DP magnitudes.

In an alternative paradigm, *f*_2_ was varied from ∼10–32 kHz and *f*_1_ set so that either *f*_2_–*f*_1_ or 2*f*_1_–*f*_2_ was always equal to 9 kHz. Propagated *f*_2_–*f*_1_ and 2*f*_1_–*f*_2_ DPs were therefore presumed to originate from a similar generation site as *f*_2_ was varied. The two DPs were characterized in separate measurements, as they required different *f*_1_ values. Figures 4(d)–4(e) show propagated DP magnitudes measured on the BM for two *f*_2_ frequencies, with *L*_2_ = 60 dB SPL and *L*_1_ varied. Propagated DPs exhibited characteristics observed in the locally generated DPs, with *f*_2_–*f*_1_ being detectable at lower stimulus levels and growing less steeply compared to 2*f*_1_*–f*_2_, which became dominant at high *L*_1_ values. However, the growth of the propagated DPs tended to be less steep than that observed for locally generated DPs. For *L*_1_ = 40–55 dB SPL, average (± SE) growth rates for *f*_2_–*f*_1_ and 2*f*_1_–*f*_2_ were 0.59 ± 0.04 and 1.40 ± 0.02 dB/dB across all frequencies and mice (*n *=* *5). Response phases were usually stable for *L*_1_ < 60 dB SPL, with phase shifts occurring at higher levels, sometimes accompanied by amplitude notches [Fig. 4(e)]. This behavior could be a feature of the nonlinearity at the more basal sites (Lukashkin *et al.*, 2002), or else could arise from interference between DPs originating from different locations. Such interference may also explain the shallower growth rates of the propagated DPs.

Figure 4(f) shows the average maximum propagated DP amplitudes observed on the BM as a function of *f*_2_, highlighting that, while *f*_2_–*f*_1_ emerged at lower stimulus levels, 2*f*_1_*–f*_2_ always became dominant at higher levels for this measurement paradigm. Maximum *f*_2_*–f*_1_ and 2*f*_1_*-f*_2_ DP magnitudes were equivalent to responses elicited by 9 kHz tones presented at ∼24 and 43 dB SPL, respectively. Though the dominance of the 2*f*_1_*–f*_2_ DP at high stimulus levels could be partly due to its relative growth pattern at the generation site [e.g., Fig. 2], lower *f*_2_*–f*_1_ magnitudes may also be attributed to the much wider *f*_2_/*f*_1_ ratios required to elicit this DP (ranging from ∼10 to 1.4 with increasing *f*_2_, compared to a range of 1.07 to 1.6 for 2*f*_1_*-f*_2_), and greater suppression by the *f*_1_ tone, which more strongly stimulated the measurement site during recordings of *f*_2_*-f*_1_. The relative levels of the propagated DPs therefore strongly depend on the stimulus paradigm and are likely influenced by factors other than the nonlinearity at the generation site.

To assess the possible perceptual relevance of the propagated DPs, one must compare their magnitudes to the displacements elicited at the threshold of hearing. Behavioral hearing thresholds near 9 kHz in CBA/CaJ mice are ∼10–16 dB SPL (May *et al.*, 2006; Radziwon *et al.*, 2009), which would correspond to BM displacements of ∼0.14–0.3 nm (−17 to −10 dB re 1 nm) and TM displacements of ∼0.3–0.7 nm (−10 to −3 dB re 1 nm) at threshold. Thus, while differences in the presentation and calibration of acoustic stimuli in behavioral studies may complicate such comparisons, both *f*_2_*-f*_1_ and 2*f*_1_*-f*_2_ DPs appear large enough to at least be detectable, if not perceptually salient, over a range of stimulus levels.

## 4. Discussion

The present work demonstrates that *f*_2_-*f*_1_ is a significant DP in vibratory responses of the mouse cochlea. The data confirm recent measurements of *f*_2_–*f*_1_ DPs in motions of the OHC region in the gerbil base (Ren and He, 2020; Vavakou *et al.*, 2019), but further show that these DPs are locally transmitted to both the BM and TM, and are measurable on these structures as they propagate apically. Characteristics of local and propagated DPs were highly similar to those observed in electrical recordings from the cochlear fluids, inner hair cells, and auditory nerve in other species (Cheatham and Dallos, 1997; Gibian and Kim, 1982; Kim *et al.*, 1980). Previous findings of *f*_2_–*f*_1_ DP being small or absent in BM vibrations in some of these species could be due to the choice of stimulus parameters, measurement sensitivity, or the relative lability of *f*_2_-*f*_1_ (Cooper and Rhode, 1997).

The presence of even-order DPs indicates that an asymmetric output is produced by the underlying nonlinearity, which, for OHCs, is commonly attributed to the mechanotransducer function. This suggests that the stereociliary bundle's OP is not at the function's center, where the transducer gain is maximal, as is often claimed (Jeng *et al.*, 2021; Russell *et al.*, 1986). However, the bias need not be extreme, as OHC region DPs could be approximated using a Boltzmann function with OP set so that ∼33% of the maximum current is activated at rest. Of course, it is possible that the OHCs' mechanical nonlinearity has sources other than mechanotransduction (Santos-Sacchi, 1989). Anesthesia may also affect OHC function such that the relative levels of even- and odd-order DPs are not the same as in the awake state (Schlenther *et al.*, 2014). Nevertheless, the fact that a simple Boltzmann model reproduced behaviors of both low-frequency intermodulation DPs and high-frequency harmonics (Dewey *et al.*, 2021) suggests that it is a reasonable starting point for understanding OHC nonlinearity in mice.

Though the 2*f*_1_–*f*_2_ DP became quite large at high stimulus levels, the dominance of *f*_2_–*f*_1_ at low stimulus levels indicates that it may also impact perception. Indeed, its presence has been suggested to facilitate envelope encoding (Nuttall *et al.*, 2018) and detection of high-frequency vocalizations in mice (Portfors *et al.*, 2009). Interestingly, however, human psychophysical studies have typically found *f*_2_–*f*_1_ to become audible only at high stimulus levels (Goldstein, 1967; Humes, 1980). Though the underlying mechanical nonlinearity may be species dependent, there could be multiple sources of nonlinearity (e.g., inner hair cell) and other central factors that contribute to differences between mechanical and perceived DPs. The choice of stimulus paradigm also dramatically affects DP magnitudes, potentially complicating comparisons between studies. Whether DPs play a significant perceptual role, or if they are simply an inconsequential by-product of cochlear nonlinearity, requires further examination in humans and other species.

## Footnotes

^1^
Individual data sets and supplementary plots of all average data are available at: https://github.com/jbdewey/Dewey2022JASAEL.
